# Pancreaticobiliary Reflux with Normal and Relatively Long Common Channels Causing Cholelithiasis and Acute Pancreatitis in Children

**DOI:** 10.3390/jcm13247650

**Published:** 2024-12-16

**Authors:** Katsunori Kouchi, Ayako Takenouchi, Aki Matsuoka, Hiroko Yoshizawa, Chikako Nakata

**Affiliations:** 1 Department of Pediatric Surgery, Tokyo Women’s Medical University, Tokyo 162-8666, Japan; matsuoka.aki@twmu.ac.jp (A.M.); yoshizawa.hiroko@twmu.ac.jp (H.Y.); nakata.chikako@twmu.ac.jp (C.N.); 2 Department of Pediatric Surgery, Chiba University, Chiba 263-8522, Japan; a.takenouchi@chiba-u.jp

**Keywords:** common bile duct stone, choledocholithiasis, children, endoscopic retrograde cholangiopancreatography, pancreaticobiliary maljunction

## Abstract

**Background and Aims:** Pancreaticobiliary maljunction (PBMJ) has a long common channel (CC) that causes pancreaticobiliary reflux (PBR), which has been implicated in gallstones, cholangiocarcinoma, and pancreatitis. By contrast, PBR has occurred in cases with normal and longer CCs than normal but shorter than PBMJ. This pathophysiology has been primarily reported in adults and rarely in children. We sometimes observe this pathophysiology in children with pancreatitis and cholelithiasis. Herein, we report the clinical figures on the diagnosis of children with PBR in normal and relatively long CCs. **Patients and Methods:** This study included seven children who complained of refractory pancreatitis and cholelithiasis diagnosed with PBR in normal and relatively long CCs at our institution from August 2018 to September 2024. We measured the lengths of their CCs and sphincter of Oddi muscles via endoscopic retrograde cholangiopancreatography (ERCP) and cholangiography. In addition, amylase and lipase levels in bile juice were measured. **Results:** All seven children demonstrated elevated amylase and lipase levels in bile juice obtained from gallbladder drainage and/or the common bile duct. ERCP and cholangiography indicated 2.2–5.5-mm lengths of CCs in their cases, which are normal lengths in two cases and relatively longer (0.3–1.1 mm: mean 0.6 mm) than the normal upper limit of CC in children in five cases, with their CCs shorter than the sphincters of Oddi. All children underwent extrahepatic bile duct resection and bilio–jejunal anastomosis, demonstrating no clinical symptom recurrence postoperatively. **Conclusions:** Some children with cholelithiasis and pancreatitis exhibit normal and relatively long CCs, causing PBR and inducing gastrointestinal diseases. Careful investigation by ERCP and cholangiography focused on the CC length, and pancreatic enzyme level assessments in bile juice are useful for diagnosing PBR in children with cholelithiasis and refractory pancreatitis.

## 1. Introduction

Common channel (CC), which opens into the duodenal papilla, is formed when the pancreatic and bile ducts merge within the duodenal walls, thereby reporting an incidence of CC formation of 55–82% [1,2,3,4]. Its length increases with age, up to a maximum of 5 mm in children [5]. Recent studies have revealed that adults with a relatively long CC develop pancreaticobiliary reflux (PBR), although the sphincter of Oddi (SO) surrounds the CC in normal gastrointestinal morphology [6,7]. This pathophysiology is an intermediate union between normal and pancreaticobiliary maljunction (PBMJ) and is redefined as a high confluence of pancreaticobiliary ducts (HCPBD) in adult cases [8]. PBR frequently induces acute pancreatitis, cholelithiasis, and gallbladder cancer in patients with HCPBD [6,7,9]. Furthermore, other studies indicate that PBR occurs in patients with a morphologically normal ductal system [9,10,11,12,13,14,15]. Several studies have proved this phenomenon using magnetic resonance cholangiopancreatography (MRCP) [13], endoscopic retrograde cholangiopancreatography (ERCP) [10,11,12], and cholangiography [14]. Thus, PBR has occurred even in adults with normal or intermediate pancreaticobiliary junction (PBJ), although reports in children are rare. In the pediatric cases, several investigators have reported markedly elevated pancreatic enzyme levels in bile juice from choledochal cysts and PBMJ without common bile duct dilatation [16,17,18]. However, the measurement of pancreatic enzymes in bile in pediatric cholelithiasis of unknown cause and recurrent acute pancreatitis (RAP) has not received much attention. We have been investigating PBJ morphology and diseases using MRCP [19] and ERCP [20,21,22,23] in children. Throughout this process, we have actively evaluated pancreatic enzyme levels in bile juice, as we believe that PBR may be responsible for various conditions, such as cholelithiasis and RAP. We have identified seven pediatric cases with normal and intermediate union of PBJ, those who complained of PBR-induced recurrent pancreatitis and cholelithiasis. The detailed observation of PBJ and the evaluation of pancreatic enzyme levels in bile juice are important in understanding the pathophysiology in pediatric cases with cholelithiasis and acute pancreatitis but without bile and pancreatic ducts comorbidities or dilatation. Herein, we report the clinical figures on PBR diagnosis in children with normal and intermediate union of PBJ.

## 2. Materials and Methods

### 2.1. Patients

Children suspected of having bile duct or pancreatic disease underwent serum liver and pancreatic enzyme tests, as well as ultrasonography (US). Further investigations, including MRCP and ERCP, were performed in accordance with our criteria. Inclusion criteria: Cholelithiasis and/or choledocholithiasis without comorbidities such as hemolytic disease, hereditary erythrocytosis, RAP, common bile duct stricture of unknown cause, or suspected sphincter of Oddi dysfunction (SOD). Exclusion criteria: Choledochal cyst, cholelithiasis and/or choledocholithiasis with clear comorbidities, or RAP due to medication or other obvious causes. When the children underwent surgery, intraoperative cholangiography was performed via the gallbladder. A total of seven pediatric cases were diagnosed with PBR occurring with normal and intermediate pathophysiology of PBJ at our institution from August 2018 to September 2024, and Table 1 summarizes their characteristics. The chief complaint was cholelithiasis, RAP, jaundice, and recurrent abdominal pain in three, two, one, and one cases, respectively.

### 2.2. Methods

MRCP was performed as previously described [19]. ERCP was conducted under general anesthesia with a PJF 240 duodenoscope (Olympus, Tokyo, Japan) in infants and a JF260v duodenovideoscope (Olympus) in older children. The PJF 240, with a video system, has a narrow tip diameter of 8.8 mm, making it suitable for ERCP in infants [23]. Intraoperative cholangiography was conducted by directly puncturing the gallbladder and infusing contrast medium. The length of the CC was assessed by ERCP and cholangiography, and SO movement was observed on sequential videos. The duct that demonstrated contractile motility was considered an SO segment, and its length was measured. SO manometry was performed as previously described by Guelrud et al. [24]. A 4-F double-lumen catheter was used to measure basal sphincter pressure by three pull-throughs via the main papilla. A 4-F endoscopic nasobiliary drainage (ENBD) tube and gallbladder drainage were used in four and two patients, respectively. Amylase and lipase levels in the bile juice obtained via the ENBD tube, gallbladder drainage, ERCP catheter, and intraoperative puncture of the gallbladder were investigated. The Ethics Committee of Tokyo Women’s Medical University (approval number: 5728, 10 December 2021)) approved this study, conducted under the 1964 Declaration of Helsinki (revised in 2013).

### 2.3. Levels of Evidence

The articles reviewed for this report were selected and classified according to five levels of evidence, as previously described [25] and listed as follows: (1) Level I: meta-analysis of double-blind randomized clinical trials; (2) Level II: cohort non-blinded studies and non-randomized clinical trials; (3) Level III: good quality case-control studies and non-randomized cohort studies; (4) Level IV: case series and poor quality case-control studies; and (5) Level V: case report articles and expert opinions.

## 3. Results

### 3.1. Cases

#### 3.1.1. Case 1

**Figure 1:**
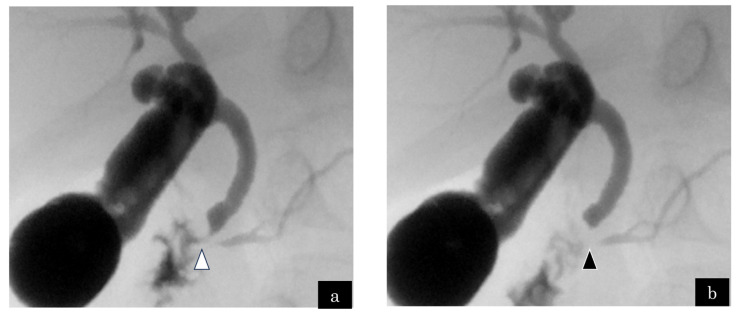
Case 1: A 2-year-old boy who complained of recurrent acute pancreatitis. (**a**) The common channel (white arrowhead) was observed on endoscopic retrograde cholangiopancreatography via the main papilla. (**b**) The communication between the bile and pancreatic ducts was interrupted by the construction of the sphincter of Oddi (black arrowhead). The length of the common channel and sphincter of Oddi segment were 3.5 and 6.3 mm, respectively.

A 2-year-old boy presented with RAP. Genetic analysis indicated trisomy 13. MRCP demonstrated no abnormalities in the bile and pancreatic ducts, whereas ERCP exhibited a relatively long CC. Cholangiography revealed 3.5 and 6.3 mm lengths of the CC and SO segments, respectively. Communication between the bile and pancreatic ducts was interrupted during SO contraction (Figure 1a,b). Lipase was 768 IU/L in bile juice obtained via gallbladder intraoperatively. We considered PBR as a factor in the onset of RAP. The patient underwent bilio–jejunal anastomosis and was symptom-free postoperatively.

#### 3.1.2. Case 2

A 10-year-old girl presented with jaundice and abdominal pain. She reported a family history of Von Hippel–Lindau disease. Blood examination revealed increased liver enzyme and bilirubin levels (aspartate transaminase: 116 U/L, alanine transaminase: 139 U/L, direct bilirubin: 7.5 mg/dL, and amylase: 53 U/L). US and MRCP demonstrated dilatation from the intrahepatic bile duct to the middle of the common bile duct (CBD); however, the duodenal site of CBD was not dilated. ERCP revealed middle CBD stenosis, and the lengths of CC and SO segments were 4.9 and 8.8 mm, respectively. Communication between the bile and pancreatic ducts was interrupted during SO contraction. Furthermore, the patient demonstrated obstructive jaundice. Therefore, an ENBD tube was inserted into the hepatic bile duct, passing through the CBD stenosis. Bile juice obtained from the ENBD tube contained high pancreatic enzyme concentrations (amylase 9629 U/L and lipase 25,849 U/L). The patient underwent CBD excision and hepatic duct and jejunum anastomosis. The pathologic assessment of the stenotic bile duct revealed fibrous tissue proliferation and eosinophil infiltration. The patient was symptom-free postoperatively.

#### 3.1.3. Case 3

A 13-year-old boy presented with RAP from 3 years of age which occurred once or twice. He was admitted to our hospital at 13 years of age. MRCP revealed no bile and pancreatic duct abnormalities. However, ERCP and cholangiography revealed that the lengths of the CC and SO segments were 5.3 and 12.0 mm, respectively. The lipase was 1768 IU/L in the bile juice obtained via ERCP catheter. The CBD was excised, and a bilio–jejunal anastomosis was established. RAP was no longer observed postoperatively.

#### 3.1.4. Case 4

A 13-year-old boy presented with recurrent abdominal pain. Blood examination indicated normal hepatic and pancreatic enzyme levels. US demonstrated a relatively dilated gallbladder and abdominal pain at the location. Hepatobiliary scintigraphy exhibited a normal gallbladder ejection fraction. MRCP demonstrated no bile or pancreatic duct abnormalities. ERCP showed that CC and SO segments were 5.5 and 9.2 mm, respectively. Herein, the hepatic and pancreatic enzymes were not elevated during RAP. Therefore, SOD and the performed SO manometry were suspected to cause abdominal pain in the patient. The basal SO pressure was 57 mmHg. We inserted ENBD and obtained bile juice after the manometric evaluation. The pancreatic enzymes obtained from ENBD were remarkably high: amylase of 14,750 U/L, pancreatic amylase of 14,100 U/L, and lipase of 56,450 U/L. The patient’s abdominal pain was caused by PBR and SOD based on these examinations. The patient underwent bilio–jejunal anastomosis and endoscopic sphincterotomy (EST) and was asymptomatic postoperatively.

#### 3.1.5. Case 5

A 1-year-old girl presented with fever and malaise. US and CT revealed cholelithiasis and a swollen gallbladder. Antibiotic therapy was administered for cholecystitis. ERCP revealed that the lengths of the CC and SO were 3.6 and 6.0 mm, respectively. An ENBD tube was inserted to evaluate for PBR, and lipase from bile juice was measured. Lipase was measured five times, each of which demonstrated high values (542–18,580 IU/L). We suspected PBR-induced cholelithiasis, and thus, the patient underwent bilio–jejunal anastomosis. The patient became symptom-free postoperatively.

#### 3.1.6. Case 6

**Figure 2:**
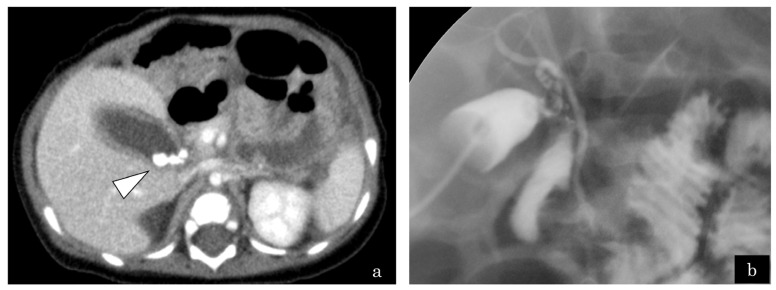
Case 6: A 4-month-old girl presented with vomiting and revealed gallbladder stones on ultrasonography. (**a**) Enhanced computed tomography revealed multiple small gallstones and gallbladder swelling (white arrowhead). (**b**) The patient developed septic shock due to cholangitis. Emergency gallbladder drainage was performed. No communication between bile and pancreatic duct was observed by cholangiography.

**Figure 3:**
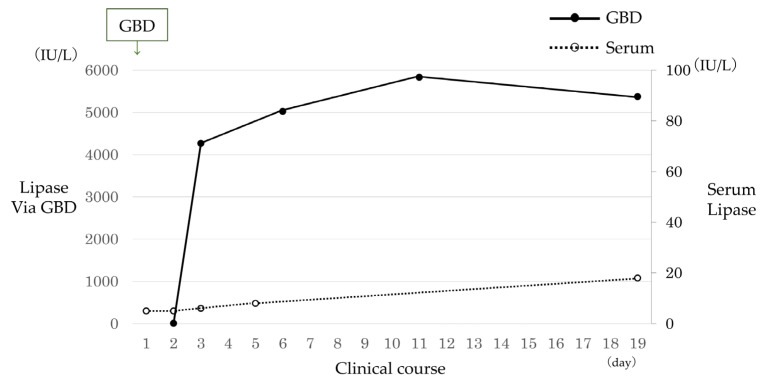
Case 6: Lipase level in serum and bile juice obtained via gallbladder drainage. Abbreviation: GBD, gallbladder drainage.

A 4-month-old girl presented with vomiting, and US revealed gallbladder stones. Computed tomography demonstrated multiple gallbladder stones and gallbladder wall thickening (Figure 2a). The patient presented with shock, thereby prompting emergency gallbladder drainage. Intraoperative cholangiography demonstrated no communication between the bile and pancreatic ducts (Figure 2b). Lipase in bile juice from gallbladder drainage was persistently high (Figure 3), which indicated that PBR recurred persistently. ERCP exhibited that the CC was 2.2 mm; however, the SO segment could not be measured because of the patient’s small size. She underwent bilio–jejunal anastomosis, with an uneventful clinical course postoperatively.

#### 3.1.7. Case 7

**Figure 4:**
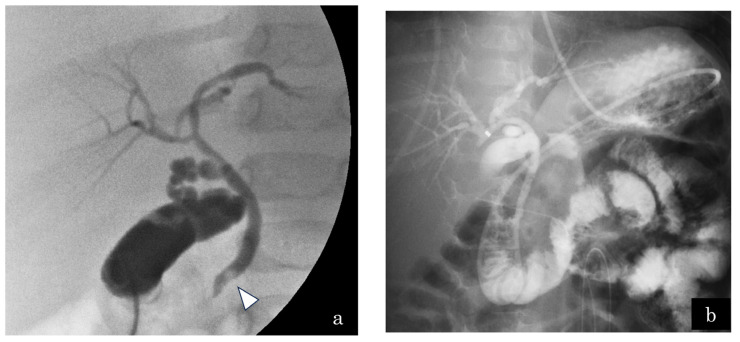
Case 7: A 5-month-old girl presented with gallbladder stones on ultrasonography. The patient developed septic shock due to cholangitis. (**a**) Emergency gallbladder drainage was performed, and a bile stone located in the distal common bile duct (white arrowhead) was observed. The pancreatic duct was not visualized by cholangiography. (**b**) A 4-Fr endoscopic nasobiliary drainage tube was inserted.

**Figure 5:**
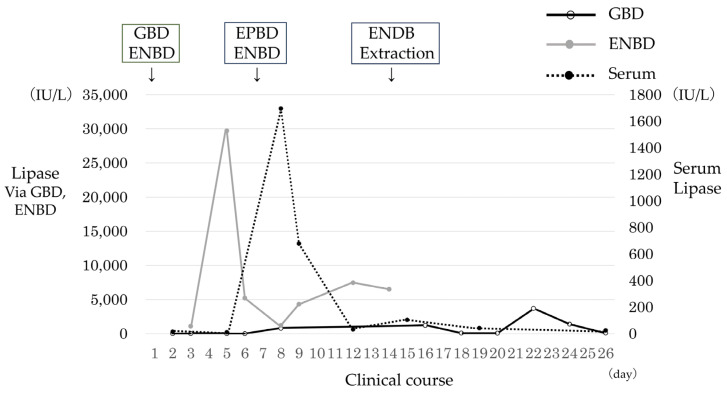
Case 7: Lipase level in serum and bile juice obtained via gallbladder drainage and endoscopic nasobiliary drainage. Abbreviations: GBD, gallbladder drainage; ENBD, endoscopic nasobiliary drainage; EPBD, endoscopic papillary balloon dilatation.

A 5-month-old girl presented with vomiting. US demonstrated multiple gallbladder stones and gallbladder wall thickening. The patient further presented with septic shock and then underwent emergency gallbladder drainage. Intraoperatively, one CBD stone was affecting the distal CBD (Figure 4a) and an ENBD tube was inserted (Figure 4b). Endoscopic papillary balloon dilatation was performed to remove the CBD stone after treating septic shock. The length of the CC was 2.4 mm; however, the SO segment could not be measured because of the patient’s small size. Lipase in the serum and bile juice obtained during gallbladder drainage and ENBD were assessed (Figure 5). The lipase level in bile juice obtained during gallbladder drainage was sometimes elevated even after ENBD removal (up to 3696 IU/L). This indicated that PBR occurred intermittently in this case, which induced gallbladder and CBD stones in the 5-month-old infant. She underwent bilio–jejunal anastomosis, with an uneventful clinical course postoperatively.

The postoperative follow-up period ranged from 12 to 95 months (mean ± SD: 62 ± 29 months). During this period, there was no recurrence of symptoms, and the postoperative course was excellent in all patients.

### 3.2. Characteristics of Seven Cases

CC and SO segment lengths demonstrated no differences between ERCP and cholangiography. Of the seven cases, CBD was dilated in only one case (Case 2), with bile duct stenosis in the middle portion. Lipase levels in bile were high in all cases, indicating that PBR was persistently or intermittently occurring. Table 2 shows the CCs of these children. Two infants (Cases 6 and 7) demonstrated a normal length of CCs, and the other five children (Cases 1–5) exhibited relatively longer lengths (0.3–1.1 mm: mean 0.6 mm) than the normal upper limit of CCs at each age of normal children, as reported by Guelrud et al. [5]. The SO segment could be measured in these five cases. All cases demonstrated a shorter CC segment than the SO segment (Table 1), indicating that the SO wrapped around the entire CC. The bilio–jejunal anastomosis was performed in all patients, with complete postoperative symptoms resolution and no pancreatitis or bile duct stone recurrence, considering that long-term PBR is a risk factor for cancer development.

Table 2 shows the normal common channel lengths of children, as reported by Guelrud et al. [5]. Seven cases of CC were compared with this normal length of CC. The difference between the measured values and the upper limit of CC for each age of children is shown.

## 4. Discussion

Cholelithiasis is less prevalent in children than in adults, with prevalence rates of 0.13–0.2% in Italy [26], 0.13% in Japan [27], and 1.9% in the Netherlands [28]. The most predominant causes of cholelithiasis are preterm birth, total parenteral nutrition, and abdominal surgery in infants. The main risk factors are hemolytic disease and hereditary erythrocytosis in children [29,30]. Obesity and oral contraceptive use have been recently determined as risk factors in adolescents [30]. The incidence of childhood pancreatitis is 3–13 cases per 100,000 persons per year compared with 5–60 cases per 100,000 persons per year in adulthood, compared with acute pancreatitis [31,32,33,34,35,36]. The risk factors of pancreatitis in children are more proportionately different than those in adults [37]. Genetic associations are >50% of risk factors for acute pancreatitis in children, followed by obstructive factors and medications [37]. The primary obstructive factor is gallstones or microlithiasis, also known as biliary pancreatitis, accounting for 3–30% of cases of acute pancreatitis in children [37]. The mixture of bile and pancreatic juice promotes gallstone and microlithiasis formation, although its contribution is very small compared with the other factors that induce gallstones and acute pancreatitis in children. Therefore, elucidating the etiology would be difficult in pediatric cases with gallstones and acute pancreatitis that do not present with a genetic predisposition or biliary or pancreatic duct dilatation.

The bile and pancreatic juice mixture is commonly observed in patients with PBMJ. Pancreatic juice refluxes into the bile duct through a long CC [2,3,6,9]. This is because pancreatic juice demonstrates greater hydropressure than bile juice. This condition is known as PBR. Pancreatic juice that drains into the bile duct causes inner lumen injury, thereby increasing the risk of gallbladder and/or bile duct cancer in adults [3,38,39]. However, PBR is not a phenomenon unique to PBMJ, but PBR also occurs in adult patients with a normal or relatively longer CC, which is longer than that in normal cases and shorter than those in cases with PBMJ (Table 3). The incidence of PBR is 5.4–25.8% in normal PBJ [10,11,12]. Both CC lengths in the presented infant cases (Cases 6 and 7) were within normal range, although measurements of pancreatic enzyme of bile juice via gallbladder drainage demonstrated PBR. SOD is considered the main factor of PBR development in normal PBJ [9,15]. Guelrud et al. [24] revealed that 18 of the 64 child cases with RAP were diagnosed with PBMJ, whereas 9 of the 18 cases with PBMJ exhibited SOD. However, studies have investigated the association between PBMJ and SOD [24,40], whereas few have explored the relationship between normal PBJ and SOD. Our study performed sphincter manometry only in Case 4, who complained of abdominal pain. We obtained a measurement of 57 mmHg and thus diagnosed the patient with SOD. Therefore, the patient underwent bilio–jejunal anastomosis and EST. The two cases presenting with RAP demonstrated no subsequent recurrence of RAP after bilio–jejunal anastomosis without EST. We then considered that SOD was not involved because only bilio–jejunal anastomosis resolved RAP in our cases.

The length of CC increases with age, up to a maximum of 5 mm in children [5]. Generally, the length of the CC in pediatric PBMJ is considered to be >5–6 mm [5,24,41]. The pediatric ERCP studies of PBMJ [5,24,42] have revealed a long CC (>10 mm). Guelrud et al. [24] indicated the CC length of PBMJ to be between 16 and 33 mm (average: 22.8 ± 5.5 mm). We focused on the length of CC and SO segments. In general, a long CC is present in cases with PBMJ, and the pancreatic and bile ducts join at a site beyond the SO action. Matsumoto et al. [43] revealed that the length of the SO segment was 12.0 ± 1.1 mm (10–16 mm) and 10.0 ± 1.5 (8–16) mm in adult patients with PBMJ and the control group, respectively. Another study in children [24] revealed that the length of the SO segment was the same as that reported by Matsumoto et al. Therefore, no significant difference was observed in the length of the SO segment between normal cases and the PBMJ group. Hence, 6–7 mm CC would be subject to the action of SO. Kamisawa [6] and Horaguchi [7] reported a significant correlation between the length of CC and biliary amylase levels (*p* < 0.01). According to Horaguchi’s study [7], biliary amylase levels in cases with a CC length > 5 mm and > 6 mm were 12,333 ± 39,956 and 17,050 ± 447,031 IU/mL, respectively. This suggests that biliary amylase levels increase with the length of the CC. Since the length of SO segment does not change depending on the pathological condition, the length of the CC and PBR are considered to be related. The discussion of the association between the length of CCs and SO is advancing in adult cases and has not been much discussed in the pediatric field. A comparison of a report of PBMJ by Guelrud et al. [5] with our cases indicated that the CC length was 6–32 mm (average: 16.0 ± 5.6) vs. 3.5–5.5 mm (average: 4.6 ± 0.9) in children older than 1 year, demonstrating that the CC length was much shorter in our cases. Their CCs were slightly longer (0.3–1.1 mm: mean 0.6 mm) than the normal upper limit of CC in children in five cases (cases 1–5; Table 2). The CC was shorter than the SO segment in five cases in which the SO segment could be measured (Table 1). Furthermore, the communication between the bile and pancreatic ducts was interrupted during SO contraction under the fluoroscopy of cholangiography or ERCP. Therefore, PBR would occur due to the slightly long CC during childhood even if the SO was wrapped around the entire CC.

Studies measuring pancreatic enzyme in bile juice [6,7,10,11,12] performed bile sampling via ERCP catheters, intraoperative gallbladder puncture, and T-tube drainage. Beltrán [15] discussed methods for diagnosing PBR in normal PBJ and believed that the most accurate method for identifying PBR is directly sampling the bile from the gallbladder during cholecystectomy before performing any manipulation in Carot’s triangle area. However, gallbladder puncture is unsuitable in patients without cholelithiasis. Moreover, Kamisawa et al. [9] predicted that PBR occurs intermittently. Therefore, we considered that a single intraoperative gallbladder puncture cannot confirm the presence of PBR. Bile could only be sampled via a catheter during ERCP or ENDB in cases without cholelithiasis. We performed several bile collections and measured the amylase and lipase levels because the catheter of ENBD insertion itself may cause PBR. We believe that repeated sampling helps reduce diagnostic errors.

The incidence of gallbladder cancer was 8.5–50% in adult cases with HCPDB, for which cholecystectomy is recommended [6,7,15,44,45]. We presume that cholecystectomy prevents future gallbladder cancer, although the reliable elimination of PBR by cholecystectomy is difficult to prove. Prolonged pancreatic fluid exposure is a risk factor for cancer development in the biliary system in children. Therefore, we selected bilio–jejunal anastomosis to completely separate the bile and pancreatic ducts. The postoperative follow-up period in our cases ranged from 12 to 95 months (mean ± SD: 62 ± 29 months). During this period, no recurrence of symptoms was observed. Based on these findings, it was concluded that cholelithiasis and pancreatitis were caused by PBR.

## 5. Limitations and the Future Perspectives of the Study

This study had some limitations. First, sphincter manometry was not conducted in all cases. Examining SO function by SO manometry in discussing the cause of PBR in cases with normal or near normal length CC will be important in the future. Second, the length of the SO segment could not be measured in infants. The fine movement of the SO could not be visualized in infants even with maximum magnification of the X-ray fluoroscopy images. Thus, we indicate assessing basal sphincter pressure and SO segment by developing a small-diameter catheter for sphincter manometry in young children. The pathophysiology of pancreaticobiliary reflux with normal and relatively long common channels is less common in children. Therefore, we hope that this study can serve as a baseline for future multicenter, large-scale studies.

## 6. Conclusions

Some children with cholelithiasis and RAP demonstrated a normal and relatively long CC, which has mainly been reported in adults. Some cholelithiasis and/or RAP cases with this pathophysiology may develop symptoms as early as infancy. Therefore, monitoring pancreatic enzyme levels in bile juice and examining the morphology of PBJ through ERCP and cholangiography is crucial for elucidating the pathophysiology of PBJ in children with cholelithiasis and RAP without comorbidities, genetic abnormalities, and biliary dilatation.

## Figures and Tables

**Table 1:** Characteristics of pediatric patients with normal and intermediate pancreaticobiliary junction.

Case	Age/Sex	Chief Complaint	Comorbidities	Length of CC/SO Segment (mm)	Dilatation of CBD
1	2 yrs/M	RAP	Trisomy 13	3.5/6.3	none
2	10 yrs/F	jaundice	VHL	4.9/8.8	Yes
					Stenosis in CBD
3	13 yrs/M	RAP	none	5.3/12.0	none
4	12 yrs/M	abdominal pain	none	5.5/9.2	none
5	1 yr/F	cholelithiasis	none	3.6/6.0	none
6	4 mos/F	cholelithiasis	none	2.2/Not measurable	none
7	5 mos/F	cholelithiasis	none	2.4/Not measurable	none
		choledocholithiasis			

Abbreviations: mos, months old; yrs, years old; M, male; F, female; CC, common channel; CBD, common bile duct; RAP, recurrent acute pancreatitis; VHL, Von Hippel–Lindau; SO segment, the sphincter of Oddi segment.

**Table 2:** Normal common channel length with age and its length in our cases.

Age (Years)	<1	1–3	4–6	7–9	10–12	13–15
Length of CC (mm; range)	1–3	1.7–2.8	2.2–3.5	2.1–3.8	3.6–4.3	3.1–4.5
Mean (mm)	1.86	2.2	2.86	3.28	3.96	4.0
Upper limit of CC (mm)	3.0	3.1	3.6	4.1	4.4	5.0
Our cases	case 6: 2.2	case 1: 3.5 (+0.4)			case 2: 4.9 (+0.5)	case 3: 5.3 (+0.3)
	case 7: 2.4	case 5: 3.6 (+0.5)			case 4: 5.5 (+1.1)	

Abbreviations: CC, common channel.

**Table 3:** Articles regarding pancreaticobiliary reflux in adult patients with normal or relatively longer common channel.

Author	Level of Evidence
Kamisawa T, et al. [6], 2010	II
Horaguchi J, et al. [7], 2014	II
Itokawa F, et al. [8], 2015	II
Kamisawa T, et al. [9], 2006	III
Sai JK, et al. [10], 2003	III
Itokawa F, et al. [11], 2004	III
Horaguchi J, et al. [12], 2008	III
Sugita R, et al. [13], 2017	III
Ueno K, et al. [14], 2015	III

## Data Availability

Data generated or analyzed during this study are available from the corresponding author by request, subject to institutional review and a data use agreement.
